# Risk factor analysis investigating the use of potentially inappropriate medications

**DOI:** 10.3389/fmed.2026.1823206

**Published:** 2026-07-08

**Authors:** Ryo Nonaka, Hatsuho Ejima, Saki Nakayama, Yuiko Suzuki, Yurika Hirota, Emiko Kurosawa, Shigeto Mashiko, Atsuhiro Kanno, Katsutoshi Furukawa

**Affiliations:** Division of Geriatric and Community Medicine, Faculty of Medicine, Tohoku Medical and Pharmaceutical University, Sendai, Japan

**Keywords:** aging, fragmented care, medical facility, medication, older adults, polypharmacy, potentially inappropriate medication

## Abstract

**Background:**

Polypharmacy, defined as the concurrent use of multiple medications, is becoming increasingly prevalent among older adults, and is strongly associated with potentially inappropriate medication (PIM) use. However, the relationship between healthcare utilization patterns and PIM use remains poorly explored. Therefore, in this study, we aimed to identify the risk factors for PIM use, focusing on age and the number of medical facilities consulted.

**Methods:**

In this multicenter, retrospective, observational study, we enrolled 351 outpatients undergoing treatment at Tohoku Medical and Pharmaceutical University Hospital or Ishinomaki Municipal Hospital. PIMs were identified using the Beers Criteria (2023). Associations were analyzed using Spearman’s correlation, Wilcoxon rank-sum test, and multiple logistic regression.

**Results:**

Age was found to be positively correlated with the number of prescribed medications (*r* = 0.476; *p* < 0.0001). Gastrointestinal medications were prescribed most frequently, while proton pump inhibitors were the most common PIMs. Patients prescribed ≥1 PIM consulted significantly more medical facilities than those without PIMs. Multivariate analysis identified age, hypertension, gastrointestinal disorders, and neuropsychiatric disorders (excluding dementia) as independent risk factors of PIM use.

**Conclusion:**

Overall, our findings showed that advanced age and fragmented healthcare utilization were significantly associated with PIM prescriptions. Strategies promoting coordinated care and medication reviews may reduce the number of inappropriate prescriptions in older adults.

## 1 Introduction

Recent trends in global population aging have led to a substantial increase in the number of older adults with multimorbidity worldwide. According to the World Health Organization, the proportion of individuals aged ≥ 65 years continues to rise worldwide (1), leading to increased healthcare and medication use.

Polypharmacy, generally defined as the concurrent use of multiple medications, is a major clinical concern in geriatric medicine (2) associated with adverse drug events, drug–drug interactions, falls, hospitalization, and mortality (3–5). The prescription of potentially inappropriate medications (PIMs), defined as drugs whose risks outweigh their benefits, is particularly concerning in older adults. The American Geriatrics Society Beer criteria (2023) are widely used to identify PIMs to guide safer prescribing practice (6).

In addition to multimorbidity, fragmented healthcare, characterized by visits to multiple medical facilities, may contribute to inappropriate prescribing practices, owing to insufficient information sharing and lack of medication reconciliation (7). Previous studies have demonstrated a positive association between the number of prescribers and polypharmacy (8). However, data regarding the relationship between the number of medical facilities consulted and PIM use remain limited (9).

In this context, the present study aimed to evaluate the association between age and medication burden and to identify the risk factors for PIM use, focusing particularly on healthcare utilization patterns among outpatients.

## 2 Materials and methods

### 2.1 Study design

This multi-center retrospective observational study enrolled 351 outpatients (147 male, 204 female) aged ≥ 18 years who consecutively visited Tohoku Medical and Pharmaceutical University Hospital or Ishinomaki municipal hospital in 2024. The mean age of the participants was 72.3 ± 17.5 (mean ± standard deviation) years (Table 1). The numbers of prescribed medications, comorbidities and medical institutions consulted were obtained from the electronic medical records. The inclusion criteria were: age ≥ 18 years and prescription of ≥1 medication in order to clarify a positive correlation between age and total numbers of medications taken. The exclusion criteria were: temporary hospital visits and terminal illness due to any disorder.

**TABLE 1:** Demographics and comorbidities of the patients.

Patient demographics	
Age, mean ± SD (years)	72.3 ± 17.5
Sex male/female	147/204
Comorbidities	
Dementia (%)	33
Hypertension (%)	62.1
Gastrointestinal disorders (%)	42.2
Dyslipidemia (%)	27.8
Diabetes (%)	40.4
Neuropsychiatric disorders (%)	30.2

### 2.2 Definition of PIMs and polypharmacy

All the medications were classified based on Anatomical Therapeutic Chemical Classification (ATC) (revised version) (10). Polypharmacy was defined as prescriptions for >4 medications (5). PIMs were extracted from the data of patients aged ≥ 65 years based on the American Geriatrics Society Beers criteria (2023) (6).

### 2.3 Statistical analyses

The association between age and the number of medications was evaluated using Spearman’s rank correlation coefficient. The Wilcoxon rank-sum test was applied to compare the total number of medical facilities consulted by patients with and without PIM use. Multiple logistic regression analysis was conducted to identify risk factors for PIM use. Statistical analyses were conducted using Statistical Analysis System version 9.4 (SAS Institute Inc., Cary, NC, United states). Continuous variables were expressed as mean ± standard deviation. Statistical significance was defined as *p* < 0.05.

### 2.4 Statement of ethics

This study adhered to the Declaration of Helsinki, and was approved by the Ethics Committee of Tohoku Medical and Pharmaceutical University Hospital. Due to the retrospective study design and complete anonymization of the data, the requirement for informed consent was waived by the ethics committee.

## 3 Results

We obtained data from 351 outpatients (147 male, 204 female) aged ≥ 18 years, and the mean age was 72.3 ± 17.5. The average number of total medications in all the patients is 7.12 ± 3.88 (mean ± SD). On the other hand, the average numbers of PIMs are 0.63 ± 0.75 and 1.18 ± 0.41 in all the patients and in the patients who take at least one PIM, respectively.

**FIGURE 1:**
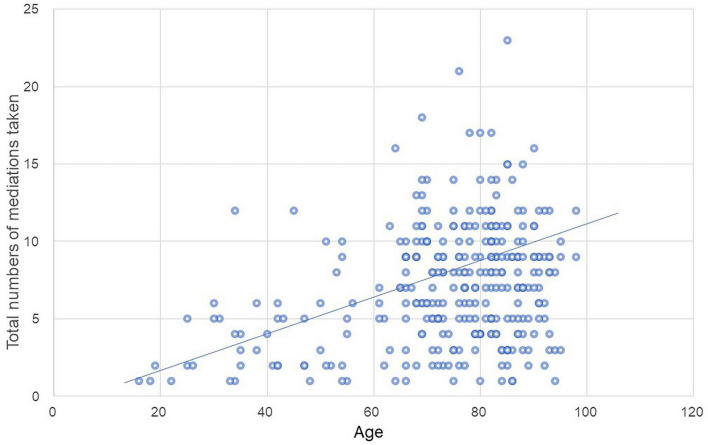
Total numbers of medications taken by each patient plotted against age, showing a positive correlation between these two factors.

**FIGURE 2:**
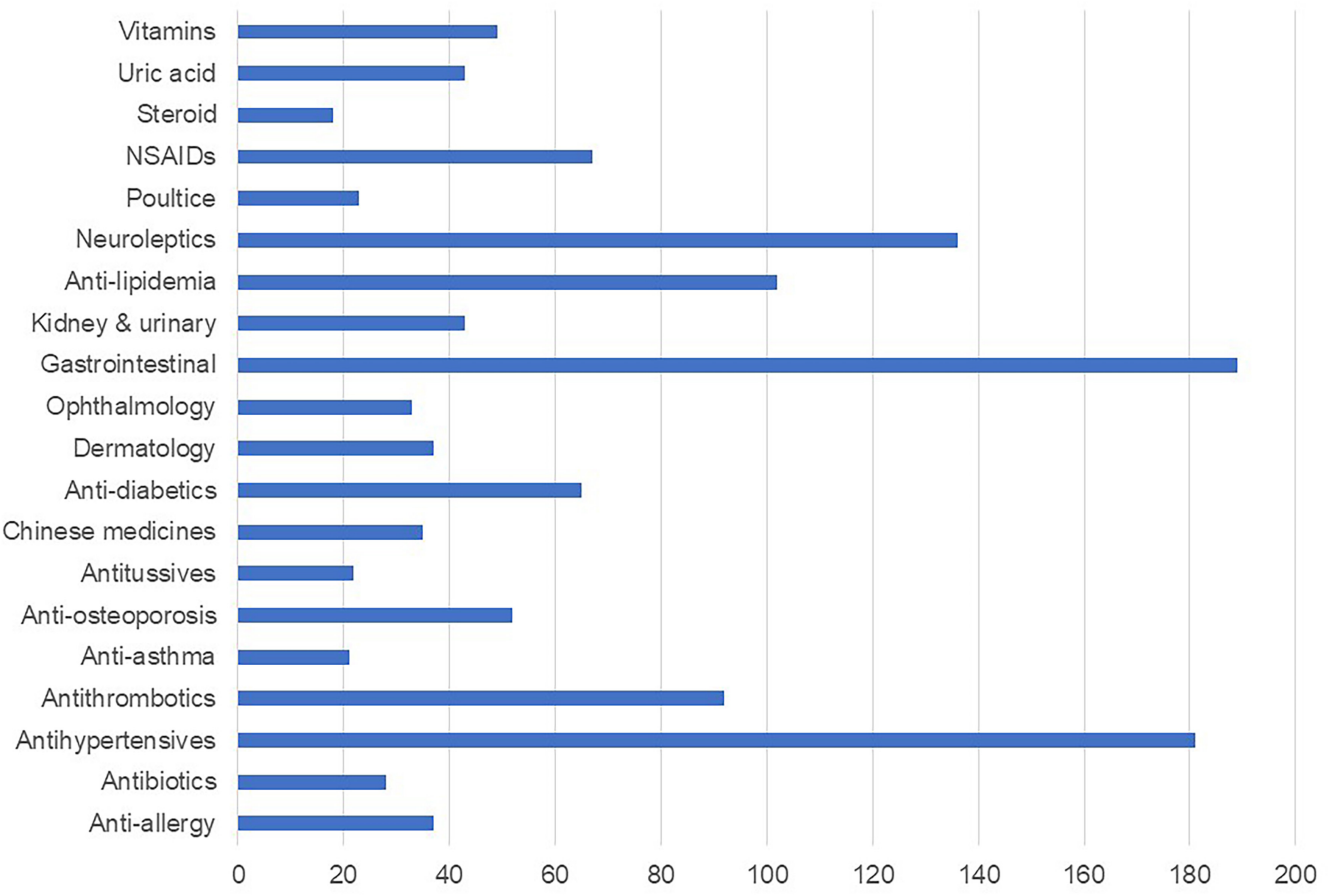
Total numbers of each different type of medication taken. Gastrointestinal medications were the most commonly prescribed, followed by antihypertensives.

**FIGURE 3:**
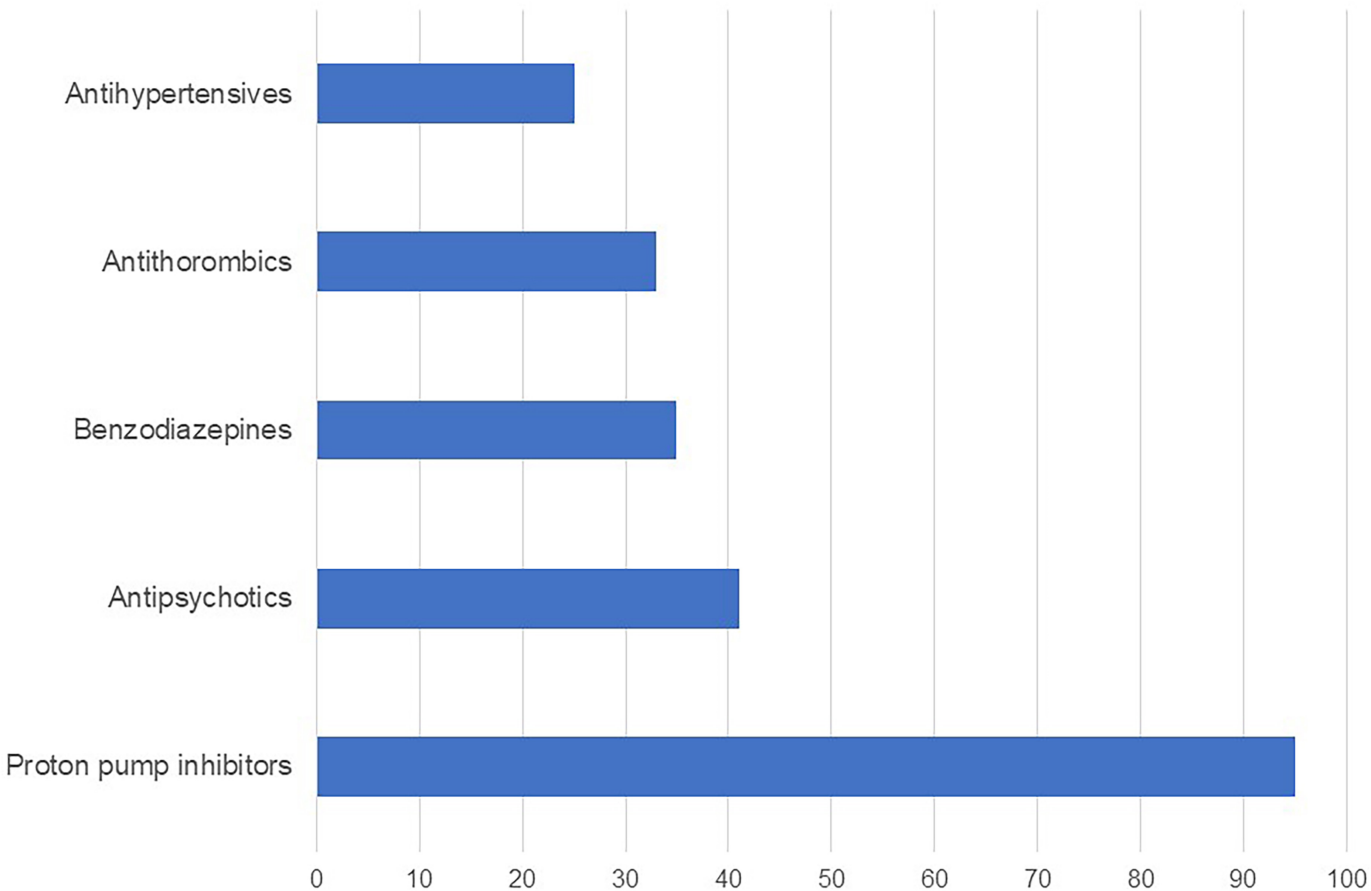
Total numbers of each poetntially inappropriate medication (PIM) category identified. Proton pump inhibitors were the most common, followed by antipsychotics.

**FIGURE 4:**
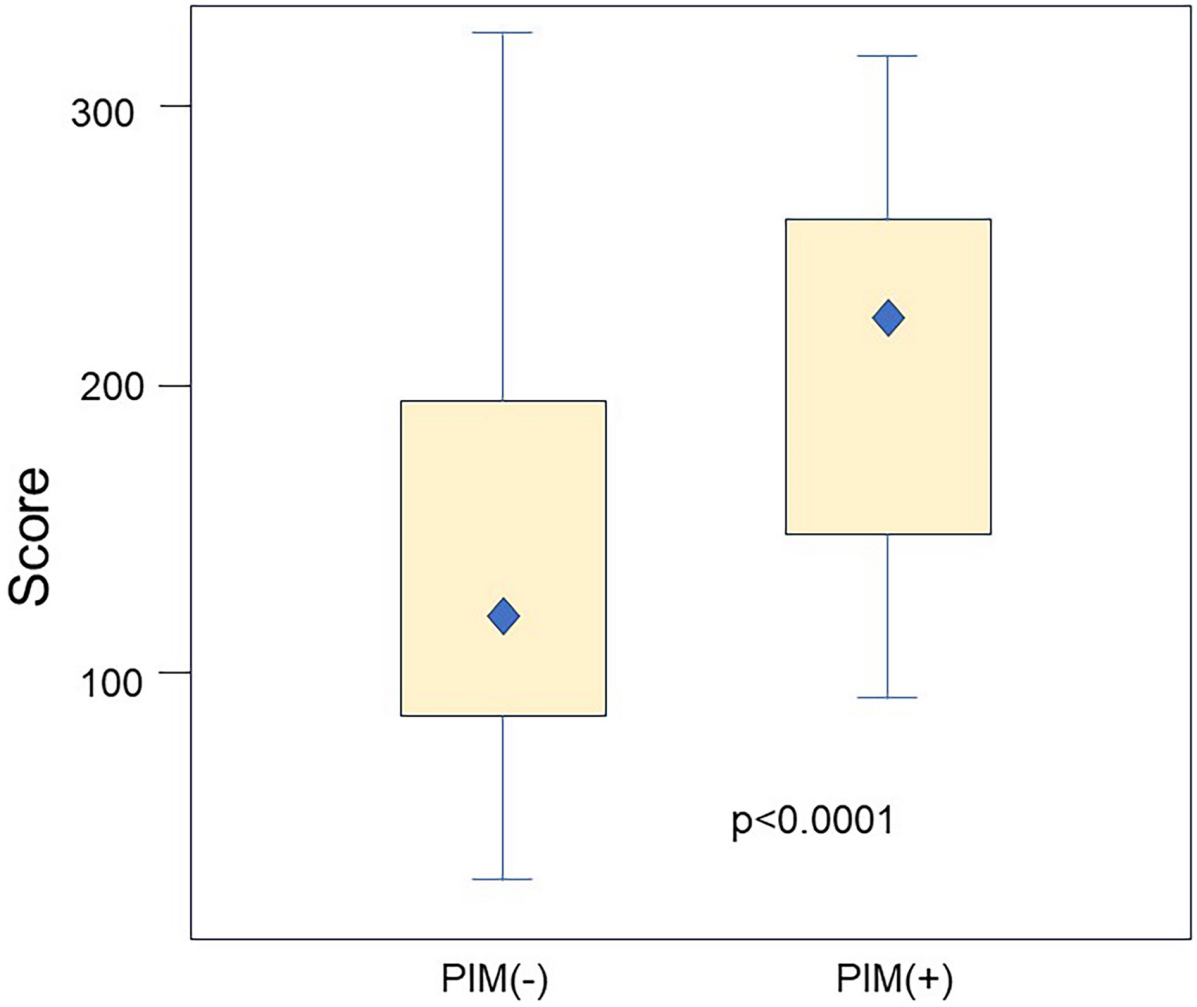
Comparison of the total number of medical facilities consulted between patients with and without prescriptions for potentially inappropriate medication (PIM). The y-axis indicates score = rank of the number of medical facilities consulted.

The association between age and number of medications in all the patients was plotted and evaluated using Spearman’s rank correlation coefficient (Figure 1). These variables indicated a strong positive correlation (correlation coefficient, 0.47557; *P* < 0.0001). The correlation between age and the number of medications was also positive (*P* = 0.038 and correlation coefficient = 0.22) in the older adult age group (≥65 years) although it was weaker than that in all the patients. Figure 2 presents the total number of medications taken, indicating that gastrointestinal medications were the most commonly prescribed, followed by antihypertensive medications. The total number of patients in each PIM category is presented in Figure 3. Proton pump inhibitors (PPIs) were found to be the most commonly used PIMs. The Wilcoxon rank-sum test was applied to analyze the relationship between the number of medical facilities consulted and PIM use (Figure 4); the results showed that patients who were prescribed at least one PIM consulted more medical facilities than those who were not significantly (*P* < 0.0001). This finding suggests a positive correlation between the number of medical facilities consulted and PIM prescriptions.

Finally, multiple logistic regression analysis was performed to elucidate the risk factors for PIM use. Age, hypertension, gastrointestinal disorders, and neuropsychiatric disorders (excluding dementia) were associated with increased PIM use, whereas dementia, dyslipidemia, and diabetes were not (Table 2).

**TABLE 2:** Results of multiple logistic regression analysis of potentially inappropriate medications (PIMs) for each risk factor.

	Estimated value	Standard error	Wald chi square	Confidential interval		*P*-value
				Lower	Upper	
Age	0.0286	0.0101	8.1173	0.008815	0.048393	**0.0044**
Dementia	−0.3856	0.2692	2.0511	−0.913237	0.142035	0.1521
Hypertension	1.0146	0.2657	14.5828	0.493823	1.536381	**0.0001**
Gastrointestinal disorders	1.7761	0.2929	36.7627	1.202025	2.350182	**<0.0001**
Dyslipidemia	−0.4585	0.2558	3.2119	−0.959863	0.042862	0.0731
Diabetes	0.336	0.2781	1.4604	−0.209076	0.881076	0.2269
Neuropsychiatric disorders (exc. dementia)	1.5525	0.2477	39.2914	1.067008	2.037991	**<0.0001**

Bold values indicate statistically significant.

## 4 Discussion

In this multicenter retrospective study, we demonstrated three major findings. First, age was found to be significantly correlated with the total number of prescribed medications. Second, gastrointestinal medications, particularly PPIs, were identified as the most commonly prescribed drugs and PIMs. Third, patients prescribed PIMs consulted a greater number of medical facilities, while fragmented healthcare was associated with inappropriate prescriptions. Fourth, age, hypertension, gastrointestinal disorders, and neuropsychiatric disorders (excluding dementia) were risk factors for PIM use.

The positive association between age and medication count identified in the present study is consistent with previous studies showing that multimorbidity increases with age, leading to a higher medication burden (3). Polypharmacy increases the risk of adverse drug events and functional decline, particularly in older adults with frailty (4).

Proton pump inhibitors were the most commonly identified PIMs. This is important, as long-term PPI use has been associated with an increased risk of fractures, *Clostridium difficile* infections, and renal dysfunction (11). Despite guideline recommendations limiting prolonged use, PPIs are commonly continued without reassessment, contributing substantially to inappropriate prescriptions (12). It was reported that PPIs were the most common and problematic PIMs in older patients (13, 14). Therefore, we consider that unnecessary PPI usage in older people should be avoided as much as we can.

Importantly, our findings highlighted the association between the number of medical facilities consulted and PIM use. Fragmented care may lead to the duplication of therapy, insufficient deprescribing, and inadequate medication reconciliation. Latimer et al. demonstrated that a higher number of prescribers was associated with inappropriate medication use, supporting our observation that care fragmentation is a critical risk factor (15). Strengthening interinstitutional communication and implementing systematic medication reviews may mitigate this issue.

Multivariate analysis identified hypertension, gastrointestinal disorders, and neuropsychiatric disorders as significant risk factors for PIM use. These conditions commonly require long-term pharmacotherapy, which increases the risk of a cumulative medication burden (16). Interestingly, dementia was not identified as an independent risk factor, possibly reflecting cautious prescription practices for patients with cognitive impairment (17). Although Salh et al. (18) reported that polypharmacy and PIM use are associated with poorer quality of life outcomes in older patients with type 2 diabetes, our findings resulted in no strong correlation between PIM use and diabetes with multiple logistic regression analysis (*P*-value: 0.2269). We speculate that this was likely because our study did not investigate their quality of life in detail. One study reported that the most frequent PIM classes were benzodiazepines, atypical antipsychotics, opioids, and non-steroidal anti-Inflammatory drugs in the emergency department (19), but our data indicated that PPIs were most common PIMs in the older patients. We consider that this difference was caused by that the study by Mousavi and Diziche (19) and ours were conducted in an emergency and chronic outpatient setting, respectively.

This study had several limitations. First, its retrospective design precludes the drawing of causal inferences. Second, all data were obtained from two Japanese institutions, which may limit its generalizability. In addition, the severity of comorbidities and functional status were not evaluated. In the future, larger, multicenter prospective studies should be conducted to overcome these limitations.

## 5 Conclusion

Advanced age and fragmented healthcare utilization are strongly associated with PIM use. Interventions, such as comprehensive geriatric assessment, deprescribing protocols, and improved care coordination, may reduce inappropriate medication use and improve patient safety in aging populations.

## Data Availability

The datasets used and/or analyzed during the current study are available from the corresponding author on reasonable request.
